# A pilot study for self-guided, active robotic training of proprioception of the upper limb in chronic stroke

**DOI:** 10.1186/s12984-025-01660-6

**Published:** 2025-06-07

**Authors:** Duncan T. Tulimieri, GilHwan Kim, Joanna E. Hoh, Fabrizio Sergi, Jennifer A. Semrau

**Affiliations:** 1 Department of Kinesiology and Applied Physiology, University of Delaware, Tower at STAR, 100 Discovery Blvd, Rm 234, Newark, DE 19713 USA; 2 Department of Mechanical Engineering, University of Delaware, Newark, USA; 3 Department of Biomedical Engineering, University of Delaware, Newark, USA

**Keywords:** Proprioception, Training, Stroke, Robotics, Sensorimotor

## Abstract

**Background:**

Proprioceptive impairments of the upper limb are common after stroke. These impairments are not typically addressed during assessment or rehabilitation. Currently, most robotic paradigms for training of the upper limb have focused solely on improving motor function or have targeted proprioception in individuals with combined use of visual feedback. Our goal was to design a training paradigm that directly targets proprioception of the upper limb, while minimizing reliance on other sensory information to improve sensorimotor function after stroke.

**Methods:**

In this pilot study, 5 individuals with stroke and 5 age-matched controls were tested on a single-day proprioceptive training paradigm. Here, participants used a joystick with their less-affected arm to send commands to a KINARM exoskeleton that would passively move their more-affected arm. To complete the passive reaching task, participants relied only on proprioceptive feedback from the more-affected arm and were only given knowledge of results information after each trial. Sensorimotor function of the upper limb was measured pre- and post-training via robotic measures of motor function [Visually Guided Reaching (VGR)] and position sense [Arm Position Matching (APM)]. Sensorimotor function was quantified as a Task Score, which incorporated multiple task-relevant parameters for both VGR and APM. Changes in sensorimotor performance due to training were calculated as the pre- to post-training difference for VGR and APM within the control and stroke groups.

**Results:**

We found significant improvements from pre-training to post-training for VGR in individuals with stroke (p < 0.001, CLES = 100) that were not observed in control participants (p = 0.87, CLES = 80). We observed significant changes from pre- to post-training in both VGR (Posture Speed, Reaction Time, Initial Direction Angle, Min–Max Speed Difference, and Movement Time) and APM (Contraction/Expansion Ratio_x_ and Shift_y_) parameters.

**Conclusions:**

Our novel proprioceptive training paradigm is one of the first to implement a self-guided sensory training protocol. We observed improvements in motor function and proprioception for individuals with stroke. This pilot study demonstrates the feasibility of self-guided proprioceptive training to improve motor and sensory function in individuals with stroke. Future studies aim to examine multi-day training to examine longer-term impacts on upper limb sensorimotor function.

## Background

Proprioception refers to our sense of static position (position sense) and movement (kinesthesia) in space [1]. Proprioception is critical for movement execution of the upper limb [2–5] and when proprioception is largely eliminated, as in individuals with sensory deafferentation, movement quality is significantly compromised [6, 7]. Neurological impairments, such as stroke, have also been shown to impair proprioception, with many individuals (~ 50 to 60%) after stroke exhibiting some type of proprioceptive impairment [8–12]. 

Historically, motor impairments following stroke have been studied extensively and remain a primary area for targeted improvement in neurorehabilitation [13–18]. In contrast, proprioceptive impairments are an often underexplored area of rehabilitation, as clinicians report that 1–25% of completed evaluations contain proprioceptive assessments [19]. Further, they report that use of evidence-based somatosensory training occurs in 18% or less of clinical interventions [20]. However, recent work has utilized robotic paradigms for the upper limb to examine whether proprioception can be improved [21–26]. Previous work in adults who are neurologically intact has shown efficacy for training proprioception using forced choice paradigms. Here, the participant’s arm is passively moved to the right or left of the midline. They report the perceived direction of movement and then receive feedback on response accuracy [21, 24, 26]. Other paradigms have required participants to make unseen reaches in 3D space to remembered targets [22] and actively retrace a pattern after being passively moved through the pattern [23]. A recent study implemented a forced choice paradigm in individuals with stroke and found improvements in both motor and proprioceptive behavior, providing promising evidence for targeted improvement of proprioception after stroke [25]. It is unsurprising that proprioceptive training improved reaching behavior as proprioception is critical for smooth and coordinated reaching movements [2, 4, 6, 27, 28]. Additionally, two of these studies have promoted active subject participation [22, 23] and yielded more robust improvements in proprioception compared to those studies with more passive designs [21, 24–26]. 

The goal of the current study was to design a proprioceptive training paradigm to improve proprioception in individuals with chronic stroke that would build on the insight gathered from a set of recent successful proprioceptive training studies [21, 23–26]. Here, we target the following task objectives: (1) promotion of active subject participation during task execution, (2) require and encourage participants to use real-time proprioception of the more-affected limb for task success, (3) cooperative interplay between the less-affected limb to actively guide passive movement of the more-affected limb. For evaluation of the developed paradigm, a sample of individuals post-stroke and control participants were recruited to participate in a single-session proprioceptive training protocol. We hypothesized that robotic proprioceptive training would result in a reduction of proprioceptive deficits associated with chronic stroke. Therefore, we predicted that (1) individuals with stroke would improve both their motor and proprioceptive performance after training, and (2) individuals with stroke would show more spatial and temporal errors and slower learning during proprioceptive training compared to age-matched controls [29]. 

## Methods

### Participants and protocol

**Fig. 1:**
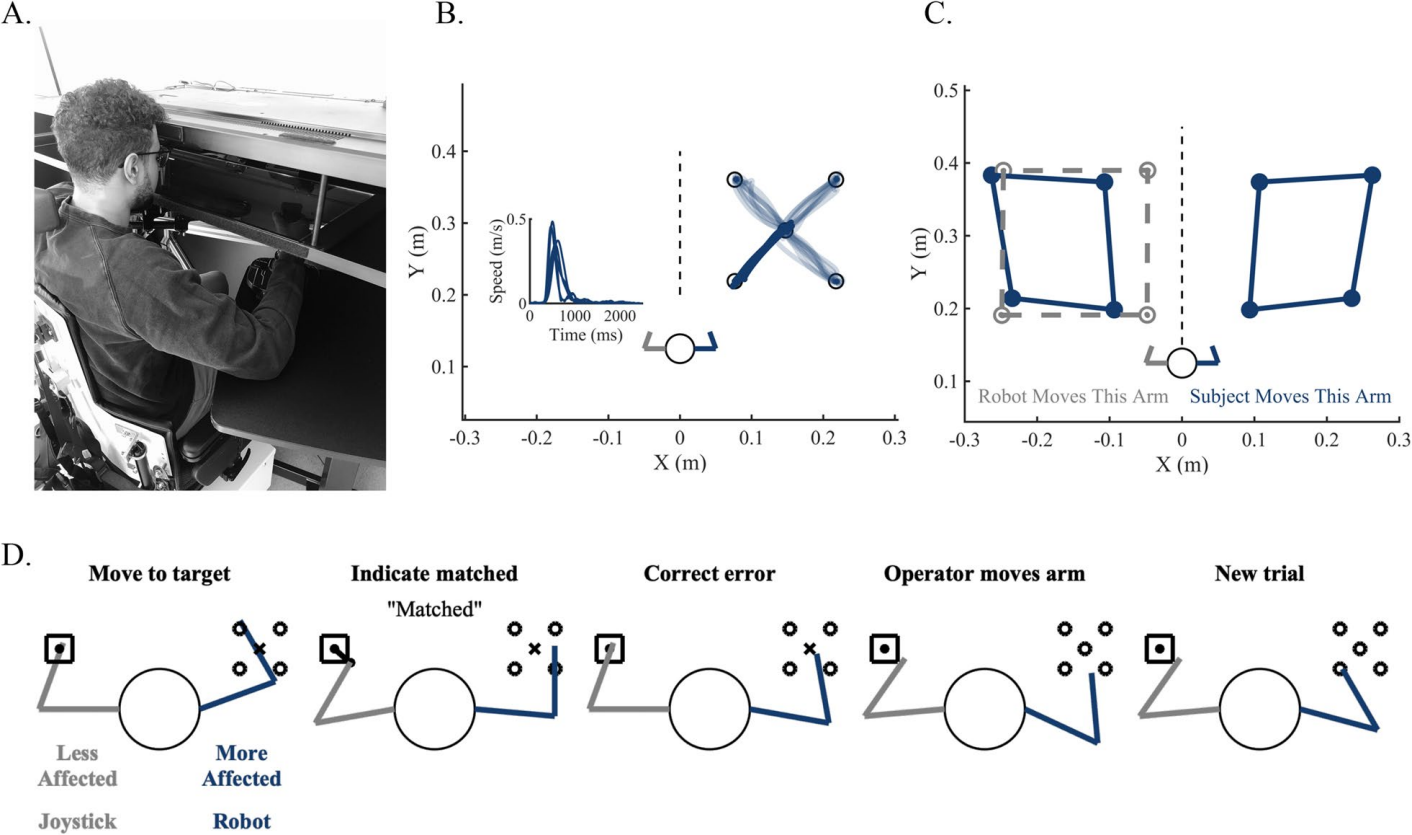
**A** Picture of the KINARM exoskeleton, integrated augmented reality display, and integrated joystick setup. Here, the participant has their left arm supported by the robot, while their right arm sits at an equal height supported by an adjustable table. The participant grips the joystick with their right hand while it is securely mounted to the table. Typically, a blocking screen covering the hands and arms, in addition to a bib around the shoulders are used to reduce visual feedback. However, for visualization of the methodology, the blocking screen has been opened to see the positioning of the participants’ right hand that is gripping the joystick. **B** Exemplar data from a control participant for the Visually Guided Reaching Task (VGR), where participants made reaching movements to each of four peripheral targets. The speed traces correspond to participant reaching performance to the bottom left-hand target. **C** Exemplar data from a control participant for the Arm Position Matching Task (APM), where for this subject the robot passively moved the left arm and the participant mirror-matched the end-point position of the robot with the right arm. Note, this task is completed in the complete absence of visual feedback of the limb. **D** Depiction of a single trial progression of the Proprioceptive Training Task. Here, in ”Move to Target”, a target is shown in the workspace on the side of the more-affected limb. Using the joystick that is gripped by the less-affected limb, the participant uses the joystick to engage passive control of the more-affected limb to align the more-affected limb with the target position. The participant then reports to the operator “Matched”, indicating that they feel that they have aligned their more–affected limb with the target. After the participant has indicated that they have matched the target, the robot passively moves the less-affected limb to correct residual error (“Correct Error”) and to passively align the participant with the end target. The operator then passively moves the more-affected limb to a pseudo-randomized starting location to eliminate memory of presented targets (“Operator Moves Arm”), after which a “New Trial” begins

A total of 10 participants (age-matched control: N = 5 and individuals with stroke: N = 5) participated in a pilot training study using the KINARM Exoskeleton and a joystick (Fig. 1A) [30]. The following inclusion criteria were used for all participants: normal or corrected-to-normal vision and at least 18 years of age. Individuals with stroke were included if they had a single, unilateral, chronic stroke (> 6 months post-stroke). The following exclusion criteria were used for all participants: previous recent history of significant upper body injury, history of a disease that may impact sensation (e.g., diabetic sensory neuropathy), and any history of a neurological disease or injury (e.g., Parkinson’s disease) other than stroke (Table 1). The current study was approved by the University of Delaware Institutional Review Board and all participants provided informed consent.

**Table 1:** Participant demographics

	Age-matched control (n = 5)	Individuals with Stroke (n = 5)
Age—mean ± std	64 ± 6	69 ± 9
Sex	2 M, 3 F	2 M, 3 F
Dominant hand	4 R, 1 L, 0 A	5 R, 0 L, 0 A
Months post-stroke		81 [18, 100]
More affected side		4 R, 1 L
FM-UE (maximum = 66)		43 [17, 65]
FIM (maximum = 126)		125 [121, 126]
TLT {0, 1, 2, 3}		3, 1, 1, 0
PPB		2.0 [0, 10]
BIT (maximum = 146)		143.2 ± 3.03
Field cut		None
MoCA (maximum = 30)		26.6 ± 1.67

Values presented next to field name indicate scoring categories (TLT) or maximum value

*M* Male, *F* Female, *R* Right, *L* Left, *A* Ambidextrous, *FM-UE* Fugl-Meyer Upper-Extremity Assessment, *FIM* Functional Independence Measure, *TLT* Thumb Localization Test, *PPB* Purdue Pegboard, *BIT* Behavioral Inattention Test, *MoCA* Montreal Cognitive Assessment. For age, BIT, and MoCA, values are reported as mean ± standard deviation. For month post-stroke, FM-UE, FIM, and PPB, values are reported

### General robotic methods

Participants were seated in the KINARM exoskeleton with their shoulders at ~80° abduction. The lengths of the robot arm segments were adjusted to fit each participants limb length. Participants were then wheeled into the integrated augmented reality system to begin testing. For Pre- and Post-Assessments (see below), participants were seated with both arms supported by the KINARM. For the Training Protocol (see below), participants were seated with one arm in the KINARM and the opposite arm supported by a small table with the joystick secured to the table. This table was adjusted to be at the same height as the opposite arm that was supported by the KINARM (Fig. 1A). 

### Clinical assessments

For participants with stroke, we used the following clinical measures to characterize upper limb function: the Upper Extremity portion of the Fugl-Meyer Assessment (FM-UE) to examine motor function of the upper limb [31], the Functional Independence Measure (FIM) to determine functional ability [32], the Thumb Localizer Test (TLT) to determine upper limb position sense status [33], the Purdue Pegboard (PPB) to evaluate upper limb and hand dexterity [34], the Behavioral Inattention Test (BIT) to screen for visuospatial neglect [35], and the Montreal Cognitive Assessment (MoCA) to screen for cognitive impairment [36]. Participants with stroke also completed visual field confrontation testing to determine if visual field cuts were present (Table 1). 

### Robotic pre- and post-assessments

To assess the effects of training, we used two KINARM robotic tasks known to quantify upper limb motor control (Visually Guided Reaching (VGR), Fig. 1B) and upper limb position sense (Arm Position Matching (APM), Fig. 1C). The methods for both tasks have been previously described in detail [8, 9, 37–39]. During the VGR task, participants were instructed to make reaches to a visual target that would appear on the screen [39]. Briefly, a 1 cm red target would appear on the screen at one of four locations. Participants were instructed to move their arm, where their fingertip was represented as a 1 cm white cursor, to the target and hold until the next target appeared. Participants started each trial at the center of the four locations. All participants performed VGR with both arms. During the APM task, all visual feedback of limb location was eliminated. Without vision of their arms, the robot would passively move one arm to one of four positions within the workspace and participants would actively move their arm to mirror-match the final position of their passively moved arm [8]. For this task, individuals with stroke had their more-affected arm passively moved by the robot and actively matched the movement location of the robot with their less-affected arm. The limb that was passively moved was counterbalanced for age-matched controls.

### Training protocol

The objective of each training trial was for participants to use a joystick to actively guide their opposite arm to a target position. More specifically, the joystick manipulations the participants made with their less-affected arm were translated to passive movement imposed by the robot on the opposite side. Three different end target locations and four different start positions for each end target (total of 12 starting positions) were used in the robotic training session. The start positions for each target set were defined as the vertices of a square with a 20 cm edge and centered around the target location (Fig. 1D). Before the training trials started, participants were allowed four familiarization trials with the joystick and KINARM set up. Here participants were allowed to use the joystick to control the position of their hand inside the KINARM to ensure they understood the relationship between the operation of the two devices. At the beginning of each training trial, visual information about the target hand location and current hand location was provided via a cyan and white circle with a diameter of 1 cm, respectively. When the visual target appeared on the screen, the participant then used the joystick to guide passive movement of their opposite arm that was supported by the robot to the seen position of the end target. Visual cursor information for hand position was extinguished 500 ms after the start of the trial, but the visual target remained on the screen to avoid memory confounds. After the participant verbally confirmed that they felt they reached the end target, the trial ended. After each trial ended, participants were passively moved to the next (pseudorandomized) starting position by the experimenter who would use the joystick to guide the arm to the next starting location. In this mode, visual information about target and current hand location was provided to begin the next trial. The starting position sequence was pseudorandomized over a list of four starting positions for each target in order to make it difficult for participants to perform the task by only refining their action plan that involved their less-affected arm. In that case, in fact, participants would be able to achieve task success by only recalling the exact sequence of actions to be implemented with their less-affected arm to reach the target position and without having a reliance on online proprioceptive feedback.

The experimenter-controlled joystick was implemented to (1) reduce potential proprioceptive drift in individuals with stroke, and (2) ensure uniform starting positions across participants. Participants completed 9 trials from each start position, for a total of 108 trials. There were 4 unique trajectories per target set, for a total of 12 unique trajectories overall. Overall, the training took 38 ± 5 min for participants with stroke and 31 ± 4 min for control participants.

### Implementation of joystick input to control the robotic arm

The joystick signal was processed and sent to the robot computer using Simulink (Mathworks, Natick, MA, USA). The joystick signal (two 14-bit signals for x and y) was converted in a normalized range ([−1, 1]), and processed to ensure a continuous and smooth movement trajectory command signal fed to the KINARM. The joystick signal with magnitude smaller than 0.01 was filtered out using a deadband filter to eliminate noise. The filtered joystick input command was then scaled to the amount of force applied to a virtual mass-damper system (gain:1.8E-5 N). The virtual mass-damper system had a mass of 1.25 kg and damping constant of 5 Ns/m in both the x and y direction. Via a Simulink transfer function model, we calculated the velocity of the virtual mass every 1 ms, subject to the force input extracted by the processed joystick signal. The resulting velocity signals in each x- and y-direction were filtered using a moving average filter with a 10-sample window. The filtered velocity signal was then sent from the computer running Simulink to the robot computer, which directly controlled the motors of the KINARM Exoskeleton via a Simulink Real-Time model, via UDP communication every 10 ms. To mitigate dramatic velocity command changes resulting from sampling ratio discrepancy between UDP communication (0.1 kHz) and Simulink Real-Time Model (4 kHz), a Kalman filter was implemented in the Simulink Real-Time Model. This filter estimates missing velocity commands between the velocity commands received via UDP communication.

### Robotic parameters to quantify behavior

Both assessment tasks (VGR and APM) are standardized and thus have auto-generated parameters and reports. To quantify motor (VGR) and proprioceptive (APM) function, for each task we utilized a composite Task Score, as well as kinematic parameters to quantify behavior. The Task Score is a single-value composite measure of all movement parameters for the task. This value can be z-transformed to compare performance of an individual against a normative model controlling for age, sex, and handedness [40]. The use of these measures has been previously described [41–47]. In addition to the Task Score, the following parameters were used to quantify upper limb motor function with the VGR task and APM task (Table 2). All parameters from the VGR and APM tasks are reported as z-transformed parameters. Therefore, performance is considered typical with scores ranging from −1.96 to + 1.96, and performance outside of these bounds are considered atypical, controlling for age, sex, and handedness [40, 48]. For one parameter (posture speed), we were unable to calculate pre-testing posture speed values for 2 participants due to extremely poor limb stabilization. These participants were excluded from this analysis due to inability to quantify posture speed at rest.

**Table 2:** Robotic parameters to quantify behavior

	Parameter	Outcome measurement
Visually Guided Reaching (VGR) parameters	Posture speed	Median hand speed when hand is resting
	Reaction time	Amount of time between end target onset and movement onset
	Initial direction angle	Angular deviation between vector from hand position at movement onset to end target and vector from hand position at movement onset and hand position after initial phase of movement (e.g., movement onset to first local minimum after max speed)
	Initial distance ratio	Ratio of distance between hand position at movement onset and offset and distance covered during initial phase of movement
	Speed maxima count	Number of hand speed maxima between movement onset and offset
	Min–max speed difference	Average difference between pairs of local speed minima and maxima
	Movement time	Time between movement onset and movement offset
	Path length ratio	Ratio of straight line from hand position at movement onset and offset and actual path travelled from movement onset to offset
	Max speed	Maximum hand speed between movement onset and offset
	Number of no reaction times	Number of trials when no movement onset was calculated
	Number of no initial stabilizations	Number of trials when participant did not stabilize in the start target
	Number of false starts	Number of trials when movement onset occurred less than 130 ms after end target turned on
	Number of no movement ends	Number of trials when movement offset was not detected before trial end
	Number of end targets not reached	Number of trials when the end target was not reached
Arm Position Matching (APM) paraments	Absolute Error_x_	Average absolute distance in x-dimension between passive hand position and mirror-reflected active hand position
	Absolute Error_y_	Average absolute distance in y-dimension between passive hand position and mirror-reflected active hand position
	Absolute Error_xy_	Average absolute distance in the xy plane between passive hand position and mirror-reflected active hand position
	Variability_x_	Average standard deviation of hand position in x-dimension for all targets
	Variability_y_	Average standard deviation of hand position in y-dimension for all targets
	Variability_xy_	Average standard deviation of hand position in the xy plane for all targets
	Contraction/expansion ratio_x_	Ratio of absolute difference between average x-position of left targets and right targets between active and passive arms
	Contraction/expansion ratio_y_	Ratio of absolute difference between average position of average y-position of left targets and right targets between active and passive arms
	Contraction/expansion ratio_xy_	Ratio of area moved between passive and active arm, and negative values for medial shift)
	Shift_x_	Average difference between mirror x-position of active arm and x-position of passive arm (positive values are lateral shift
	Shift_y_	Average difference between mirror y-position of active arm and y-position of passive arm (positive values for distal shift and negative values for proximal shift)
	Shift_xy_	Root-sum-squares of shift in the x-dimension and shift in the y-dimension

Parameters for Visually Guided Reaching (VGR) and Arm Position Matching (APM) tasks, and how these outcomes are measured

### Data and statistical analysis

To determine if sensorimotor learning occurred during the robotic training task, we first calculated and then fit trial time and end point error to a three-parameter exponential decay model. Trial time was defined as the time from the start of the trial to when the participant verbally announced they felt matched. End point error was defined as the Euclidean distance from the position when the participant verbally announced they felt matched to the desired position. We fit these data for each participant with an exponential decay model with three free parameters: Initial Error (${E}_{0}$), Learning Rate ($\lambda$), and Asymptotic Error (${E}_{n}$), as a function of Trial Number (*t*).

$$Learning \, value\left( t \right) = E_{0} *e^{ - \lambda *t} + E_{n}$$

This model fit was bootstrapped to improve estimation of fit parameters. To bootstrap this estimation, for 1,000,000 iterations, we re-sampled the data with replacement and fit the model. The bootstrapped estimation was then determined as the median from each bootstrapped-parameter-distribution.

To test our predictions, for each participant group, we compared Task Scores from the pre- and post-training time periods for both VGR and APM. We used directional permutation tests (${H}_{0}:post>pre$) with 1,000,000 permutations for these comparisons, such that we expected participants to decrease their Task Score (i.e., improve behavior) after training [49]. To quantify the effect size, we used common language effect size (CLES) which describes how often a sample from one distribution will be greater than a sample from another distribution [50]. We then performed this same analysis on each parameter for both tasks. For example, for the VGR task, we compared performance for Posture Speed from the pre- and post-training assessments, and for the APM task, we compared performance for Absolute Error_x_ during the pre- and post-training assessments. Additionally, we compared each of the three free parameters from the modified exponential decay model between individuals with stroke and age-matched controls. We used directional permutation tests for these comparisons, such that we expected individuals with stroke to show larger initial and final error as well as smaller learning rates [49]. The effect size of these comparisons was also quantified with CLES [50].

## Results

### Effects of training on reaching behavior

**Fig. 2:**
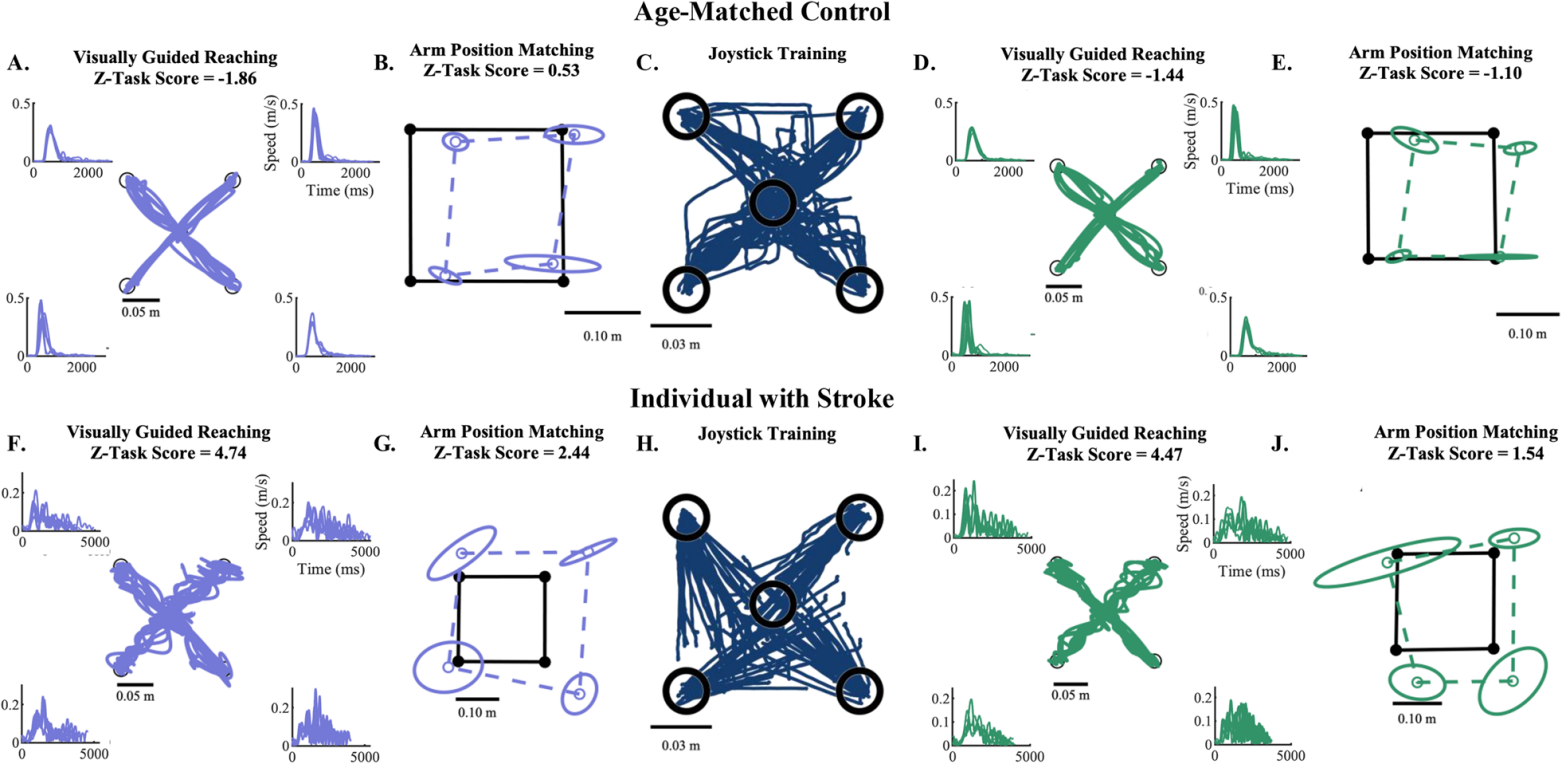
Exemplar data from an age-matched control participant (**A**–**E**) and a participant with stroke (**F**–**J**). In each row, the displayed exemplar data coordinates with the progression of testing within the study. Initial pre-testing was completed using the Visually Guided Reaching Task (VGR) and the Arm Position Matching Task (APM), as depicted in panel A and B for the control participant and F and G for the participant with stroke. Here, we observe that the participant with stroke has poorer quality of movement (**F**) and increased position matching errors (**G**) compared to the control participant (**A**, **B**). Participants then underwent the training task (C—Control, H—Stroke). Here, we observed that training accuracy was worse in the participant with stroke (**H**) as indicated by splayed end-point trajectories that do not reach the intended end target. In comparison, we see that the end-point trajectories for the control participant (**C**) typically end in one of the target locations. During post-testing, we observe similar performance for VGR and APM for the control participant (**D**, **E**) and slight improvements in VGR and a return to normal (Z-Task Score < 1.96) for APM for the participant with stroke

**Fig. 3:**
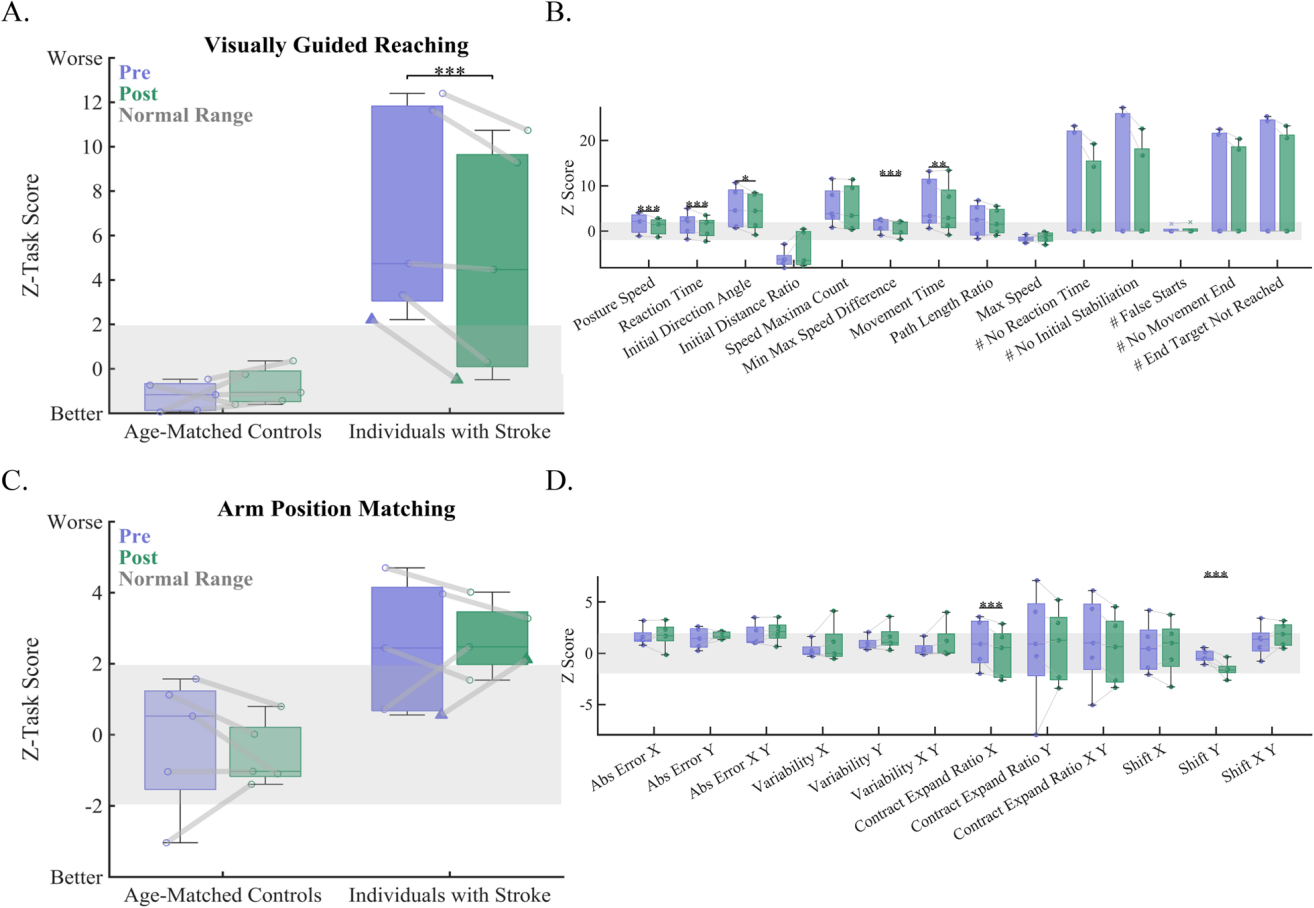
Comparisons of pre- and post-training behavior for reaching and position sense. **A** We observed that individuals with stroke significantly improved overall behavior on the Visually Guided Reaching task (Task Score), which was not observed in control participants (stroke: p < 0.001, CLES = 100.00, controls: p = 0.87, CLES = 80.00). **B** For individuals with stroke, we examined pre- and post-training behavior on individual kinematic parameters to determine what aspects of motor performance showed marked improvement. We observed significant improvements in 5 of 12 parameters for the VGR task (Posture Speed (p < 0.001, CLES = 100.00), Reaction Time (p < 0.001, CLES = 100.00), Initial Direction Angle (p = 0.016, CLES = 80.00), Min–Max Speed Difference (p < 0.001, CLES = 100.00), and Movement Time (p = 0.002, CLES = 80.00). **C** We observed that, on average both groups improved Arm Positing Matching performance (Task Score) from pre- to post-training; however, this comparison was not significant for participants with stroke or controls. **D** Examining individual parameters for the APM task for the stroke group found that individuals with stroke showed significant improvements on two APM parameters: Contraction/Expansion_x_ (p < 0.001, CLES = 100.00), and Shift_y_ (p < 0.001, CLES = 100.00). Markers represent individual participants. Triangle marker for individuals post-stroke indicates one participant with ipsilesional deficits as measured by the VGR task

To test our prediction that individuals with stroke would improve their reaching behavior post-training, we compared VGR Task Scores from pre- and post-training (Figs. 2, 3A). We found that individuals with stroke significantly improved their reaching performance (VGR Task Score Pre-Training: 6.9 ± 4.8 and VGR Task Score Post-Training: 4.9 ± 5.1; p < 0.001, CLES = 100). In contrast, we did not observe a similar improvement for age-matched controls (VGR Task Score Pre-Training: −1.2 ± 0.7 and VGR Task Score Post-Training: −0.8 ± 0.8; p = 0.87, CLES = 80). To better understand what aspects of the movements improved to drive this difference for individuals with stroke, we compared all z-transformed movement parameters. Here, we found significant improvements for five parameters: Posture Speed (VGR Score Pre-Training: 1.7 ± 2.6 and VGR Score Post-Training: 1 ± 2.1; p < 0.001, CLES = 100), Reaction Time (VGR Score Pre-Training: 1.6 ± 2.6 and VGR Score Post-Training: 0.9 ± 2.3; p < 0.001, CLES = 100), Initial Direction Angle (VGR Score Pre-Training: 5.1 ± 4.5 and VGR Score Post-Training: 4.3 ± 4.1; p = 0.02, CLES = 80), Min–Max Speed Difference (VGR Score Pre-Training: 1.4 ± 1.6 and VGR Score Post-Training: 0.8 ± 1.8; p < 0.001, CLES = 100), and Movement Time (VGR Score Pre-Training: 6.0 ± 5.6 and VGR Score Post-Training: 4.9 ± 5.7; p = 0.001, CLES = 80) (Fig. 3B).

### Effects of training on position matching behavior

To test our prediction that individuals with stroke would improve proprioceptive performance post-training, we compared APM Task Scores from pre- and post-training (Fig. 3C). We found that neither group demonstrated significantly improved proprioceptive performance (individuals with stroke—APM Task Score Pre-Training: 2.5 ± 1.9 and APM Task Score Post-Training: 2.7 ± 1.0; p = 0.66, CLES = 60 and age-matched controls—APM Task Score Pre-Training: −0.2 ± 1.9 and APM Task Score Post-Training: −0.5 ± 0.9; p = 0.24, CLES = 60). Similarly to VGR, we compared pre- and post-training performance on APM parameters for individuals with stroke and found significant improvements in Contraction/Expansion Ratio_x_ (APM Score Pre-Training: 1.0 ± 2.3 and APM Score Post-Training: 0.0 ± 2.4; p < 0.001, CLES = 100) and Shift_y_ (APM Score Pre-Training: −0.3 ± 0.6 and APM Score Post-Training: −1.6 ± 0.8; p < 0.001, CLES = 100) (Fig. 3D).

### Training data

**Fig. 4:**
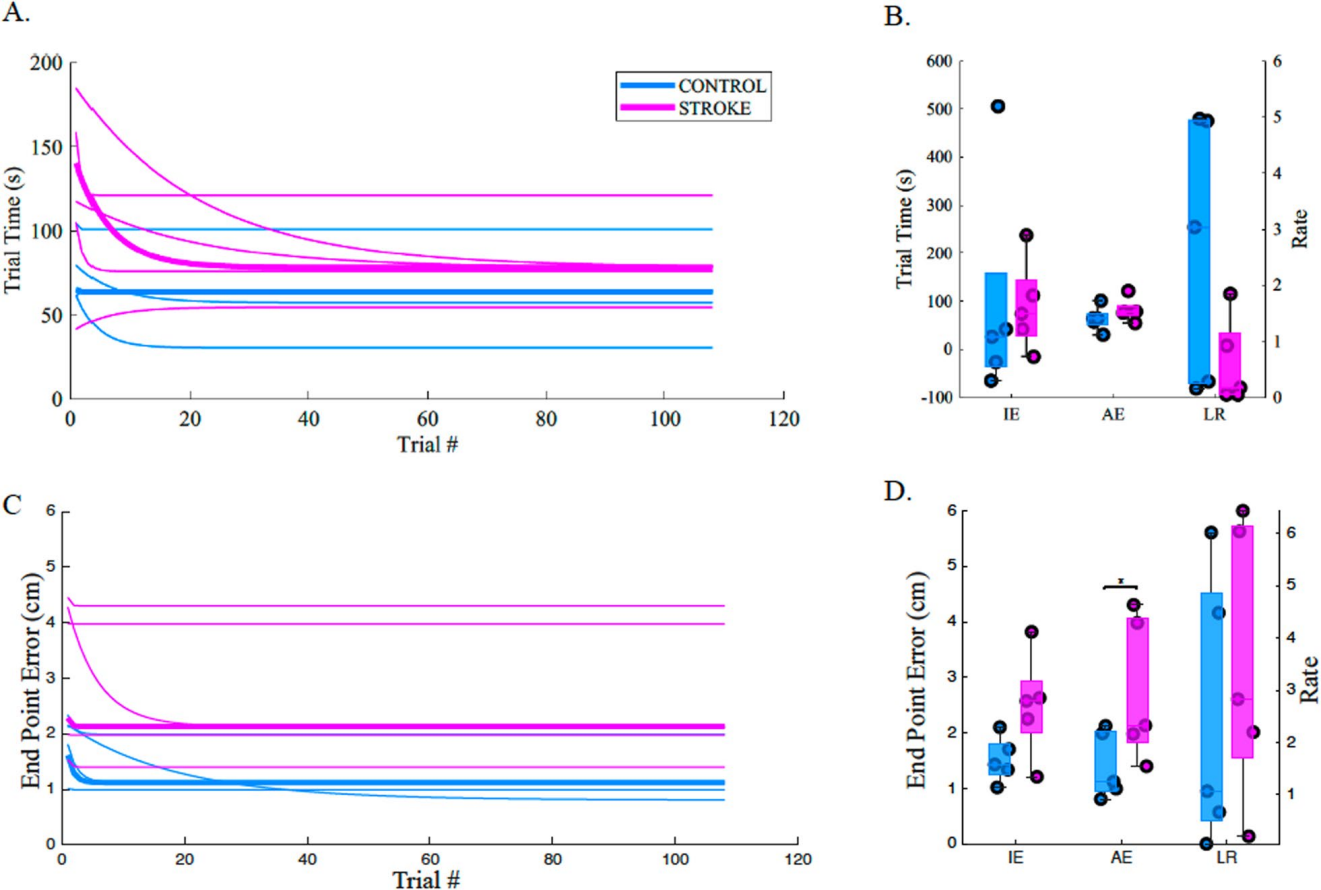
Exponential fits of learning over the course of the proprioceptive training task via Trial Time (top) and End Point Error (bottom). **A** We examined whether there were consistent decreases in trial time over the course of training. We observed that most participants (stroke and control) demonstrated reductions in how long it took to complete a trial over the course of training, with most time reductions occurring within the first 30 trials. **B** No significant differences were observed between control and stroke groups for Initial Error (IE), Asymptotic Error (AE), or Learning Rate (LR). **C** Similar to A, we observed decreases in End Point Error, with most error reductions occurring within the first 20 trials. **D** When the individual parameters were compared between groups, we found that individuals with stroke had higher End Point Error Asymptotic Error than control participants (p = 0.032, CLES = 84.00). In A and C, thin lines indicate individual participant fits, while the thicker line indicates the group median value

To test our prediction that individuals with stroke would show slower learning and more errors during training, we first bootstrapped the fits of each participant’s training data to an exponential decaying model (Eq. 1) and then compared the resulting group parameters (median of bootstrapped distribution for each participant) for both our learning metrics: trial time and end point error (Fig. 4). We found that the asymptotic value of end point error (parameter E_n_) was significantly larger for individuals with stroke compared to age-matched controls (individuals with stroke: 2.8 ± 1.3 cm and age-matched controls: 1.4 ± 0.6 cm; p = 0.03, CLES = 84).

## Discussion

We pilot tested 10 participants, 5 individuals with chronic stroke and 5 age-matched controls, on a novel proprioceptive training paradigm and assessed changes in movement and proprioception with separate standardized robotic tasks. We found that all individuals with stroke showed significant improvements in reaching behavior and most individuals with stroke showed improvements in proprioceptive behavior when comparing pre-training to post-training performance (Fig. 3). We also found that individuals with stroke did not significantly differ in their learning rate during training compared to age-matched controls (Fig. 4). Overall, our study suggests that our novel proprioceptive training paradigm may improve reaching and proprioceptive behavior in individuals with stroke.

### Targeted training approach for improving proprioception

The main purpose of the current pilot study was to develop a robotic paradigm that directly targets proprioception of the upper limb, while minimizing contributions from the visual system. Previous studies have demonstrated the efficacy of both active and passive proprioceptive training in neurologically intact participants and have shown improved proprioceptive thresholds and/or accuracy [21, 22, 51]. Other studies have examined proprioceptive training in stroke survivors and have demonstrated positive outcomes for improvements in proprioceptive function of the upper limb and/or wrist [25, 52, 53]. Notably, these previous studies in stroke have typically relied on passive movement of the limb coupled with verbal report about the status of the limb (i.e., psychometric tasks) or matched behavior with the less-affected limb. Here, we engaged participants to self-guide position of the less affected limb using a framework that seeks to reduce visual confounds and increase participant engagement within the proprioceptive training paradigm [54]. Previous work has demonstrated that with reduced explicit feedback (e.g., vision), self-guided learning effectively engages robust training patterns [55] and improved proprioceptive accuracy in older adults [56] to the benefit of motor performance. Within the objectives outlined as part of this study, we aimed to (1) promote active subject participation, (2) encourage use of real-time proprioception of the more-affected limb, and (3) facilitate cooperative interactions between the less- and more-affected limb for guidance of the more-affected limb. We observed that not only was this a feasible training task for individuals with stroke, but that our pilot study demonstrates effectiveness of these methods for improving motor performance as well as some aspects of limb position sense, suggesting that this training method is likely effective for improving upper limb function after stroke.

### Consistent improvements in reaching behavior

One of the most robust findings from this study was that all our individuals with stroke were able to improve their reaching performance after training (Fig. 3A). Not only did we observe a significant improvement in the Task Score, but we also observed improvements at the individual parameter level. Interestingly, we observed improvements in each “main” domain of movement as quantified in a previous study: upper limb postural control, reaction time, feed-forward control, and feedback control indicating that the proprioceptive training also improved many domains and components of reaching through improvements in proprioception [39] (Fig. 3B). This finding is in agreement with previous literature that has observed improvements in reaching behavior after proprioceptive training [57] and serves to further support the critical role proprioception plays in the generation of smooth, coordinated reaching movements [2, 4, 6, 27, 28]. Given that these improvements were observed in a small, but heterogenous sample, this technique provides a promising avenue for training of upper limb function after stroke across a broad spectrum of impairment levels.

### Less consistent improvements in position matching behavior

We found that most, but not all, of our individuals with stroke were able to improve their position matching behavior (Fig. 3C). This result is consistent with previous literature that found that most, but not all, individuals with chronic stroke showed proprioceptive improvements post-stroke [25]. While nearly all of our participants showed improvements in the Task Score for APM from pre- to post-training, group results for this measure were not significant. However, when we examined individual parameters, we found that the individuals with stroke significantly improved for two APM measures from pre- to post-training for Contraction/Expansion Ratio_x_ and Shift_y_. Additionally, it should be considered that the training task is both movement and position based. While we did not observe improvements on a position based proprioceptive (i.e., position sense) measurement, we would expect to see improvements on a movement based proprioceptive (i.e., kinesthesia) measurement given results from previous studies in this population [25]. Finally, it is important to note that one of our individuals with stroke exhibited ipsilateral motor deficits on the VGR task. Some individuals with stroke exhibit ipsilateral movement deficits which become apparent during online corrections of reaching movements [58]. Therefore, for this individual, their APM task performance may look impaired, but this is likely due to combined impairments in ipsilateral proprioceptive and motor function. In fact, this was one of the two individuals with stroke that demonstrated worse performance after training suggesting that their “decrease” in performance after training may be a result of impaired reaching (triangle, Fig. 3).

### Learning during training

Surprisingly, we found few differences between learning parameters for individuals with stroke and age-matched controls (Fig. 4). We believe that this was mostly due to our protocol, mainly the incorporation of familiarization trials before training trials. In typical motor learning studies, participants are not familiarized with the perturbation before they need to adapt, and learning is often driven by both the sudden onset and large initial errors of the perturbation [59]. For our design, before participants were trained, they underwent four familiarization trials where they saw the cursor that represented their fingertip and were able to use the joystick to move the cursor and their arm. Thus, participants were able to learn the mapping of joystick command to robotic passive movement during this time. We believe that without these trials, we would have seen much larger errors, in both training time and end point error, at the beginning of training for both groups, but especially individuals with stroke. This in turn would have increased the initial error term and consequently the rate term as the asymptotic learning values would most likely remain similar. Despite not observing differences in learning rate, we did observe significant differences in end point error asymptotic error between groups, where individuals with stroke typically had a higher end point error asymptotic error than age-matched control participants. This difference suggests that while the initial learning process appears to be similar between the two groups, the amount of learning or completeness of learning may be diminished in individuals with stroke which is in partial agreement with recent work that observed both slower adaptation rates and less complete sensorimotor adaptation in individuals with stroke [29, 60, 61].

### Limitations

We recognize that this study was done with a relatively small sample size (n = 5). However, the intended purpose of the current pilot study was to test the feasibility of a combined joystick and robot task in controls and individuals post-stroke. Additionally, while our intention was to recruit 5 age-matched controls for this pilot study, we must note that recruited adults who were neurologically intact had comparable, but not completely, matched ages to our cohort of individuals with stroke. We believe that the current results are promising in terms of the ability to improve sensorimotor function after stroke and require a larger sample size for more complete validation. Further, the study was conducted within a single day. Ideally, post-test assessments should be completed at least 24 hours post-training to determine greater long-term effectiveness of training. Additionally, a visual component was an element of the design of the task. Yet visual feedback in this task was only presented after trial completion and used to help the participant assess their own performance and recalibrate the mapping between their own proprioception sensation and the “true” value of their arm position. In any case, reliance on visual feedback could pose an issue for some individuals with stroke who are unable to integrate visual and proprioceptive signals [43, 62]. However, in comparison to previous proprioceptive training tasks, we believe that we minimized the inclusion of visual information as best as possible, while still allowing participants to be exposed to a task where they can have some degree of success and remain motivated. Additionally, previous work has demonstrated that ipsilateral motor deficits occur in 37% of individuals with subacute stroke and 14% of individuals with chronic stroke, which must be considered as a potential limitation when considering how to utilize this protocol in future studies as it requires bilateral interactions between the arms [63, 64]. While individuals in this study were screened for ipsilateral motor impairment prior to training, all included participants had normal clinical motor function of the ipsilesional arm (ipsilesional FM-UE = 66/66). Future studies using such techniques will need to comprehensively screen for motor status of the ipsilesional arm to ensure proper guidance of the more-affected arm. Finally, in order to evaluate true effectivity of training, a long-term, multi-day training protocol needs to be conducted. Future studies will focus on implementing this protocol for a multi-week training to evaluate long-term effectiveness of our task design.

## Conclusions

We designed a novel proprioceptive training paradigm for individuals with chronic stroke and tested the effects of this training with standardized tasks for 10 participants. This paradigm has shown to be effective at improving reaching and position matching behavior for several individuals with chronic stroke. We found that participants in all groups were able to learn during the training task. This work has implications for neurorehabilitation of individuals with chronic stoke because it demonstrates that proprioceptive training using participant-driven passive guidance of the more-affected limb can improve not only motor behavior, but also proprioception.

## Data Availability

No datasets were generated or analysed during the current study.

## Abbreviations

KINARM: Kinesiological instrument for normal and altered reaching movements
VGR: Visually guided reaching
APM: Arm position matching
CLES: Common language effect size
